# Cardiac overscreening hip fracture patients

**DOI:** 10.1007/s00402-019-03270-z

**Published:** 2019-08-31

**Authors:** S. J. M. Smeets, B. P. W. van Wunnik, M. Poeze, G. D. Slooter, J. P. A. M. Verbruggen

**Affiliations:** 1 Department of Surgery, Flevoziekenhuis, Hospitaalweg 1, 1315 RA Almere, The Netherlands; 2 Department of Surgery, Beatrixziekenhuis, Banneweg 57, 4204 AA Gorinchem, The Netherlands; 3 Department of Surgery, Maastricht University Medical Centre, Maastricht, The Netherlands; 4 Department of Surgery, Máxima Medical Center, De Run 4600, 5504 DB Veldhoven, The Netherlands

**Keywords:** Hip fracture, Cardiac screening, Preoperative screening, Geriatric, ACC/AHA guideline

## Abstract

**Background:**

The aim of this study was to prospectively investigate the adherence to the American College of Cardiology (ACC) and the American Heart Association guidelines for perioperative assessment of patients with hip fracture in daily clinical practice and how this might affect outcome.

**Methods:**

This prospective cohort study from Maastricht University Medical Centre included 166 hip fracture patients within a 3-year inclusion period. The preoperative cardiac screening and adherence to the ACC/AHA guideline were analyzed. Cardiac risk was classified as low, intermediate and high risk. Secondary outcome measurements were delay to surgery, perioperative complications and in-hospital, 30-day, 1-year and 2-year mortality.

**Results:**

According to the ACC/AHA guideline, 87% of patients received correct preoperative cardiac screening. The most important reason for incorrect preoperative cardiac screening was overscreening (> 90%). Multivariate analysis showed that a cardiac consultation (*p* = 0.003) and overscreening (*p* = 0.02) as significant predictors for increased delay to surgery, while age, sex, previous cardiac history and preoperative mobility were not. High risk patients had in comparison with low risk patients a significantly higher relative risk ratio for in-hospital mortality (RR 6, 95% CI 2–17). Multivariate analysis showed that a previous cardiac history and increased delay to surgery were predictors for early mortality. High age and previous cardiac history were risk factors for late mortality.

**Conclusion:**

Preoperative cardiac screening for hip fracture patients in adherence to the ACC/AHA guideline is associated with a diminished use of preoperative resources. Overscreening leads to greater delay to surgery, which poses a risk for perioperative complications and early mortality.

**Level of evidence:**

II.

## Introduction

Hip fractures are one of the most common orthopaedic causes leading to hospital admission in the geriatric population and are associated with high morbidity and mortality rates [1]. All hip fracture patients receive preoperative screening for perioperative risk assessment, usually by the anesthesiologist. Preoperative screening includes often a preoperative cardiac consultation. The reason for this is not cardiac clearance, but cardiac risk assessment to determinate changes in perioperative patient management, including anesthesia, pharmacological and perioperative monitoring [2]. Preoperative cardiac consultation in patients with hip fractures is often time consuming and may lead to delay to surgery. Early operative treatment within 24–48 h is advocated to minimize the potential morbidity/mortality associated with delay to surgery [3–5]. Therefore, extended cardiac evaluation should be restricted if it is unlikely to change perioperative patient management [6–9].

According to the American College of Cardiology and the American Heart Association (ACC/AHA) guidelines hip surgery is considered intermediate risk surgery, due to the quantity of hemodynamic stress it induces [2]. This means that major ischemic cardiac complications occur in less than 5% of the time. However, the overall incidence of perioperative myocardial ischemia in elderly patients undergoing hip fracture surgery has been reported to be 22–53% [10, 11]. In addition, previous studies indicated that the principal causes of in-hospital death after hip fracture were cardiac failure and myocardial infarction, occurring early after the fracture [12, 13].

To reduce the risk of perioperative cardiac events the ACC/AHA have developed guidelines for preoperative risk stratification and cardiac assessment. The key points of these guidelines are summarized in an algorithm indicating the stepwise approach of patients using clinical predictors to identify their cardiac risk category.

In this study we prospectively evaluated whether the routine preoperative screening of hip fracture patients as performed in our department is in accordance with the ACC/AHA guidelines and the consequences for daily practice in preoperative management and postoperative complications in a cohort of hip fracture patients.

## Materials and methods

### Study population

This prospective study was conducted in the Maastricht University Medical Centre in the Netherlands, a level 1 trauma center. All patients with a hip fracture admitted to the emergency department were eligible for inclusion. During a 3-year period, patients of 65 years and above were included. After surgery there was a 2-year follow-up observation period or until death. Polytrauma patients, pathological hip fractures or patients with hip fractures who did not have surgery, were excluded. The department of trauma surgery and orthopaedic surgery used a protocolized treatment algorithm regarding hip replacement or internal fixation based on our National guidelines ‘Proximal femur fractures’, 2016 [14].

At admission the following score forms were recorded: the ASA (American Society of Anesthesiologists) score for physical status [15], the Barthel index for pre-injury functional evaluation [16], Metabolic Equivalent of Task (MET) score for functional capacity [17], Mini Mental State Examination (MMSE) for cognitive impairment [18] and the Palmer and Parker score for mobility [19]. Furthermore, the preoperative cardiac work-up was described on the evaluation form using the ACC/AHA guideline template. Standard work-up after admission to the emergency room consisted of a detailed history, a complete physical examination, an electrocardiography and standard biochemical and hematologic tests. During preoperative screening the anesthesiologist decided whether a cardiac consultation was necessary. Extensive cardiac evaluation consisted of an evaluation of the patients performance state, medication review, assessment of the electrocardiogram, physical examination, an echocardiography on indication and advice was given regarding the cardiac risk in relation to the intended operation and recommendations on patient management in the perioperative period. The Maastricht Ethical committee approved the waiver of the requirement to obtain a signed consent form. All the questions and score forms were taken within the presence of a first-degree family member or sometimes legal representative in the case of dementia.

### Primary outcome measurements

Our main goal was to evaluate the preoperative cardiac evaluation in daily practice, using the algorithm proposed in the ACC/AHA guideline. Clinical predictors for each risk group are shown in Table 1. ‘Correctly screened’ accounts for those patients who had surgery without cardiac consultation with a stable cardiac situation or in the absence of a cardiac history, or patients who received a cardiac consultation when indicated by the guideline. We defined two possibilities for preoperative cardiac screening that was not in line with the guidelines. ‘Underscreening’ was defined as a cardiac consultation that was indicated by the guidelines, but not executed. In other terms: preoperative cardiac screening that fell short. ‘Overscreening’ was defined as a cardiac consultation that was not indicated by the guideline, but still performed. In other terms, preoperative cardiac screening that was too extensive. Subsequently, the primary investigator (SM) analyzed the content of the preoperative cardiac consultations in relation to the patient’s medical condition and discussed this with the research group and principal investigator (VE). These additional quality measures were taken to crosscheck our interpretations of the AC/AHA guidelines.

**Table 1:** The American College of Cardiology/American Heart Association clinical predictors for each risk group

Low risk	Intermediate risk	High risk
Advanced age	Mild angina pectoris	Unstable coronary syndromes
Abnormal ECG	Prior myocardial infarction	Decompensated cardiac heart failure
Rhythm other than sinus	Compensated or prior cardiac heart failure	Significant arrhythmias
Low functional capacity	Diabetes mellitus	Severe valvular disease
History of stroke		
Uncontrolled hypertension		

### Secondary outcome measurements

Secondary outcome measurements were delay to surgery, perioperative complications and early and late mortality. We analyzed the complication rates in relation with delay to surgery. Mortality rates were recorded in-hospital and at 30-day, 1-year and 2-year interval.

### Statistical analysis

All analyses were performed with SPSS 23 statistical software for windows (SPSS Inc., Chicago, Illinois, USA). *P* < 0.05 was considered to be statistically significant. Data were presented as mean with standard deviation (SD) or as percentages when appropriate. In case of non-normal distributed, data were presented as median with interquartile range (IR). One-way ANOVA were used to compare normally distributed and the Mann Whitney *U *test for non-normally distributed continuous variables with Bonferroni correction for multiple testing. A Pearsons chi-square (*χ*^2^) test was used to investigate whether distributions of categorical variables differed from one another. We used a Kaplan–Meier survival curve to investigate the mortality rates for each of the cardiac risk groups, comparing outcome using log rank analysis. A univariate logistic analysis of the postoperative complications was performed to identify risk factors early mortality. All important variables from univariate analysis for mortality were entered in a multivariate regression analysis.

## Results

In the study period 166 consecutive patients were eligible for inclusion. Patient and operative characteristics are presented with the representative cardiac risk groups in Table 2. There were no significant differences in preoperative status concerning ASA score, the Barthel index for functional evaluation, Metabolic Equivalent of Task (MET) score for functional capacity, Mini Mental State Examination (MMSE) for cognitive impairment and the Palmer and Parker score for mobility.

**Table 2:** Patient and operation characteristics

Variables	ACC/AHA cardiac risk group			
	Low %(*n*) *n* = 108	Intermediate %(*n*) *n* = 45	High %(*n*) *n* = 13	Total %(*n*) *n* = 166
Median age (years)	85 (IR 11)	83 (IR 11)	84 (IR 6)	84 (R 65–99)
Female sex	68% (73)	67% (30)	85% (11)	69% (114)
ASA (median)	3 (IR 1)	3 (IR 0)	4 (IR 0)	3 (R 1–4)
Barthel index (median)	18 (IR 7)	17 (IR 7)	13 (IR 11)	17 (R 1–20)
Palmer and Parker score (median)	6 (IR 5)	6 (IR 5)	4 (IR 5)	6 (R 0–9)
MET (median)	4 (IR 3)	4 (IR 3)	2 (IR 3)	4 (R 0–10)
MMSE (median)	25 (IR 12)	25 (IR 12)	17 (IR 15)	21 (R 0–30)
Mobility not impaired	39% (40/102)	40% (17/43)	18% (2/11)	38% 59/156
Use of walking aids	48% (49/102)	33% (14/43)	36% (4/11)	43% 67/156
Strongly impaired/indoors	10% (10/102)	23% (10/43)	36% (4/11)	15% 24/156
Immobile – transfers	3% (3/102)	5% (2/43)	9% (1/11)	4% 6/156
Intracapsular fractures	47% (50/107)	51% (23/45)	62% (8/13)	48% (80)
Extracapsular fractures	53% (57/107)	49% (22/45)	38% (5/13)	51% (85)
General anesthesia	57% (56/99)	60% (25/42)	73% (8/11)	54% (89)
Spinal anesthesia	43% (43/99)	40% (17/42)	27% (3/11)	38% (63)
Operation time (min., mean)	69 (SD 28)	70 (SD 27)	85 (SD 25)	71 (SD 28)
Delay to surgery (h., median)	23 (IR 20)	25 (IR 17)	31 (IR 46)	27 (R 2–135)
> 24 h	44% (47/106)	53% (23/43)	62% (8/13)	47% (78)
> 48 h	10% (10/106)	5% (2/43)	38% (5/13)^*/**^	10% (17)
Hospital stay (days, median)	8 (IR 6)	10 (IR 9)	16 (IR 9)	9 (R 2–59)

*ASA* American Society of Anesthesiologists score, range 1–6; Barthel Index score for functional capacity, range 0–20, Palmer and Parker score for mobility, range 0–9, *MET *metabolic equivalent of task score for physical activity, range 0–> 10, *MMSE* mini mental state examination score for measure cognitive impairment, range 0–30, *IR* interquartile range in case of non-normal distributed data, *R* range of data, minimum–maximum, *SD* standard deviation, in case of normal distributed data

*Significant difference in compare with low risk group, *p* < 0.05

**Significant difference in compare with intermediate risk group, *p* < 0.05

In total 8% (13/166) of patients were predicted to have a high perioperative complication risk, 27% (45/166) an intermediate risk and 65% (108/166) a low risk. In 11 (6.6%) patient’s preoperative mobility scores could not be accurately determined at admittance due to cognitive impairment and unavailability of relatives to give correct information. These data were regarded as missing data, other available data of these patients were still used for analysis. There were no significant differences concerning operative characteristics between the cardiac risk groups (Table 2).

### Preoperative cardiac evaluation

According to the guideline 93% (100/108) in the low risk group, 76% (34/45) in the intermediate risk group and 85% (11/13) in the high risk group received correct preoperative cardiac screening (Table 3). Of all patients 13% (21/166) did not receive the correct preoperative screening. The main reason for this was due to overscreening in 90% of the cases (19/21); 59% (98/166) of the patients had a cardiovascular history, which did not necessarily imply active cardiac conditions. The chance to receive a cardiac consultation increased with the patient’s perioperative cardiac risk group assignment, 9%, 33% and 100% in the low, intermediate and high risk group, respectively. We found no significant differences in outcome in the overscreening group vs correctly screened patients.

**Table 3:** Preoperative cardiac evaluations

	ACC/AHA cardiac risk group			
	Low %(*n*) *n* = 108	Intermediate %(*n*) *n* = 45	High %(*n*) *n* = 13	Total %(*n*) *n* = 166
Cardiac history	28% (30)	80% (36)*	92% (12)*^/^**	47% (78)
Cardiac consultation	9% (10)	33% (15)*	100% (13)*^/^**	23% (38)
Cardiac screening in accordance with ACC/AHA guidelines	93% (100)	76% (34)*	85% (11)*	87% (145)
Overscreening	7% (8)	20% (9)	15% (2)	11% (19)
Underscreening	0	4% (2)	0	12% (2)

*Significant difference in compare with low risk group, *p* < 0.05

**Significant difference in compare with intermediate risk group, *p* < 0.05

### Delay to surgery

The median delay to surgery was 23 h (IR 20), 23 h (IR 17) and 31 h (IR 46) for the low, intermediate and high risk group, respectively. The mean delay to surgery increased by 9.0 h (SD 25–44) when patients had a cardiac consultation (*p* = 0.06). High risk patients received significantly more preoperative cardiac consultations and had more often a delay to surgery of > 48 h (*p* < 0.005 in comparison with the low risk an intermediate risk group). Multivariate analysis showed that a cardiac consultation (*p* = 0.003) and overscreening (*p* = 0.02) were significant risk factors for increased delay to surgery, while age, sex, preoperative cardiac history and preoperative mobility were not influencing delay to surgery. In univariate analysis patients with a delay to surgery of > 48 h had significantly more respiratory complications (*p* = 0.04). In multivariate analysis an increased delay to surgery was an independent predictor for in-hospital mortality (*p* = 0.03) and 30-day mortality (*p* = 0.02) independent from cardiac risk category.

### Postoperative complications

Postoperative complications are presented by cardiac risk group in Table 4. Respiratory and cardiovascular complications occurred significantly more often in the high risk group in comparison with the intermediate and low risk group (*p* < 0.02). This was related to a significantly higher risk for in-hospital death. Pulmonary complications (RR 37.95% CI 9–156; *p* < 0.0001), and cardiovascular complications (RR 9.95% CI 3–24; *p* < 0.0001) were significant risk factors for in-hospital mortality. In a multivariate analysis an increased delay to surgery was an independent risk factor for respiratory complications (*p* = 0.009). Furthermore, a multivariate regression analysis for cardiovascular complications showed that a cardiac history was a significant risk factor, but not age, sex or delay to surgery (*p* = 0.001).

**Table 4:** Complications

Complications	ACC/AHA cardiac risk group			
	Low %(*n*) *n* = 108	Intermediate %(*n*) *n* = 45	High %(*n*) *n* = 13	Total %(*n*) *n* = 166
Number of complications	1 (IR 1)	1 (IR 1)	2 (IR 2)	1 (R 0–7)
Delirium	25% (27)	20% (9)	31% (4)	24% (40)
Wound infection	6% (6)	13% (6)	0	7% (12)
Blood transfusion	43% (46)	38% (17)	61% (8)	43% (71)
Urinary tract Infection	17% (18)	16% (7)	0	15% (25)
Pressure sores	4% (4)	9% (4)	0	5% (8)
Respiratory	11% (12)	11% (5)	46% (6)*^/^**	14% (23)
Pneumonia	8% (9)	9% (4)	31% (4)*	10% (17)
Pulmonary embolism	0	0	0	0
Respiratory failure	5% (5)	7% (28)	23% (28)*	7% (11)
Gastrointestinal tract bleeding	4% (4)	2% (1)	0	3% (5)
Gastrointestinal^a^	2% (2)	2% (1)	0	2% (28)
Cardiovascular	6% (7)	31% (14)*	62% (8)*^/^**	17% (29)
Stroke	1% (1)	2% (1)	0	1% (2)
Rhythm disorders	2% (2)	13% (6)*	38% (5)*^/^**	8% (13)
AMI	2% (2)	9% (4)	15% (2)	5% (8)
Cardiac failure	5% (5)	11% (5)	38% (5)*^/^**	9% (15)
Neurological	1% (1)	2% (1)	0	1% (2)
Implant failure^b^	7% (8)	2% (1)	8% (1)	6% (10)
In-hospital mortality	6% (6/108)	9% (4/45)	31% (4/13)*^/^**	8% (14/166)

*Significant difference in compare with low risk group, *p* < 0.05

**Significant difference in compare with intermediate risk group, *p* < 0.05

^a^Postoperative ileus, extensive diarrhea, ischemia

^b^Re-operation, luxation or deep infection of the arthroplasty or fracture fixation.

Respiratory complications consist of pneumonia, pulmonary embolism and respiratory failure together. Cardiovascular complications consist of stroke, rhythm disorders, acute myocardial infarction (AMI) and cardiac failure. *IR* interquartile range in case of non-normal distributed data, *R* range of data, minimum–maximum

### Mortality

**Fig. 1:**
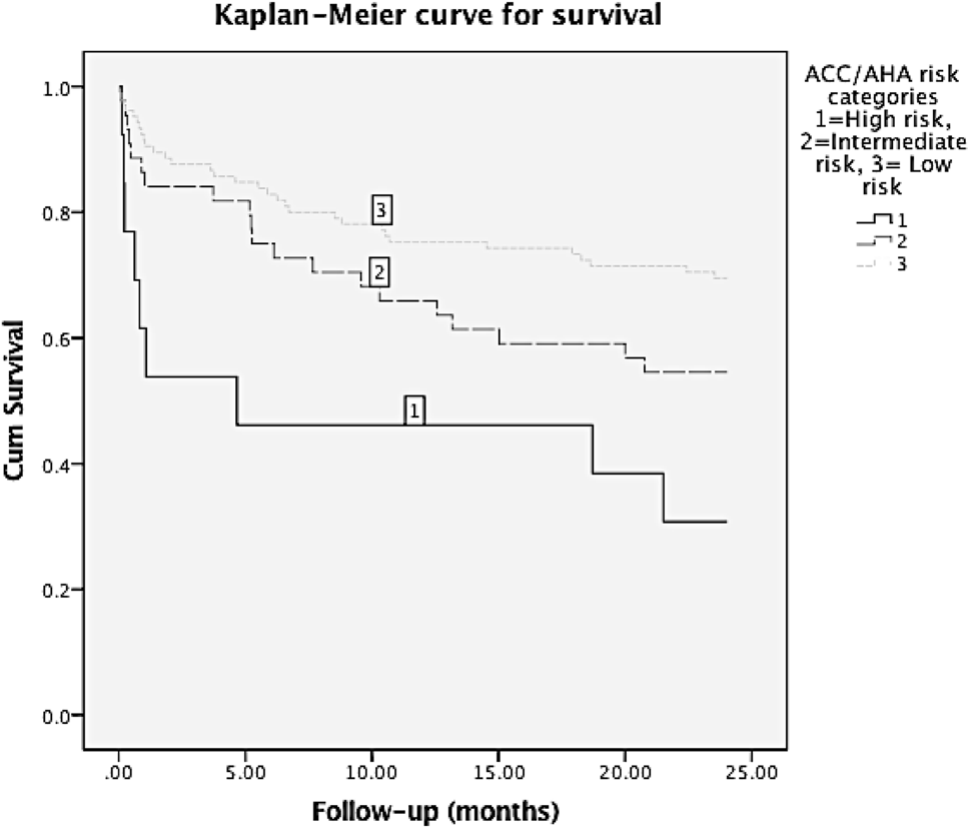
Survival curve

The mortality rates by cardiac risk group are summarized in Table 5. A Kaplan–Meier curve for survival was made with a follow up of 24 months in Fig. 1 (lost to follow-up after discharge *n* = 9). The mortality rates increased with the cardiac risk category. This led to significantly higher mortality in high risk patients for the in-hospital, 30-day, 1-year and 2-year mortality in comparison with low risk patients. High risk patients had in comparison with low risk patients a relative risk ratio (RR) of 6 (95% CI 2–17) for in-hospital mortality, RR 5 (95% CI 2–12) for 30-day mortality, RR 2 (95% CI 1.3–4) for 1-year mortality and RR 2 (95% CI 2–4) for 2-year mortality. In-hospital mortality was due to cardiovascular complications in 50% of cases, followed by respiratory failure in 36% of cases and in 14% because of other reasons.

**Table 5:** Mortality rates

Mortality	ACC/AHA cardiac risk group			
	Low % (*n*)	Intermediate % (*n*)	High % (*n*)	Total % (*n*)
In-hospital	6% (6/108)	9% (4/45)	31% (4/13)*^/^**	8% (14/166)
30-day	9% (9/102)	16% (7/43)	41% (5/12)*	13% (21/157)
1-year	25% (25/102)	37% (16/43)	58% (7/12)*	31% (48/157)
2-year	30% (31/102)	44% (19/43)	75% (9/12)*	38% (59/157)

*Significant difference in compare with low risk group, *p* < 0.05

**Significant difference in compare with intermediate risk group, *p* < 0.05

Mortality rates in relation with a prior cardiac history are presented in Table 6. These results show that a previous cardiac history is related with > 3.5 fold higher early mortality, or > 1.7 fold higher late mortality in comparison with no previous cardiac history.

**Table 6:** Mortality rates in relation to prior cardiac history

Mortality	No cardiac history % (*n*)	Cardiac history % (*n*)	*p*
In-hospital	2% (2/88)	15% (12/78)	0.002
30-day	6% (5/83)	22% (16/74)	0.004
1-year	24% (20/83)	38% (28/74)	< 0.05
2-year	28% (23/83)	49% (36/74)	0.005

Significant difference for* p* < 0.05

A Cox multiple regression analysis for mortality for the complete follow-up period of 24 months showed that high age was the only significant risk factor. With linear regression analysis we studied potential risk factors for early and late mortality (see Table 7). A cardiac history and increased delay to surgery were predictors for early mortality (in-hospital and 30-day). A high age and a cardiac history were found risk factors for late mortality.

**Table 7:** Linear regression analysis for mortality

Variables	In-hospital mortality (*p*)	30-day mortality (*p*)	1-year mortality (*p*)	2-year mortality (*p*)
Sex	0.2	0.1	0.2	0.6
Age	0.7	0.7	0.001	< 0.0001
Delay to surgery	0.03	0.02	0.1	0.2
Cardiac history	0.005	0.008	0.2	0.04

Significant difference for* p* < 0.05

## Discussion

In this prospective cohort study 87% of patients received preoperative cardiac screening in adherence to the ACC/AHA guideline. The most frequent reason for incorrect preoperative cardiac screening was overscreening (> 90%). High risk patients received significantly more preoperative cardiac consultations and experienced more often a delay to surgery of > 48 h. Multivariate analysis showed that a cardiac consultation and overscreening are significant predictors for increased delay to surgery, while age, sex, a previous cardiac history and preoperative mobility were not. Early mortality (in-hospital and 30-day) was determined by a previous cardiac history and increased delay to surgery. High age and a previous cardiac history were predictors for late mortality.

Preoperative cardiac screening for hip fracture patients has been subject to debate between surgeons and anesthesiologists. Therefore, the ACC/AHA provides guidelines for preoperative cardiac screening to minimize the risk for perioperative cardiac complications and preventing overuse of medical resources. High risk patients might benefit from delaying hip fracture surgery to optimize or stabilizing the cardiac comorbidities. Nevertheless, delaying surgery with a cardiac consultation that is not recommended or contributing could lead to worse outcome. Cardiac consultations increase delay to surgery, which is confirmed by several other studies [7, 20]. However, in this study, no significant differences in postoperative complications in the overscreening group vs correctly screened patients were found, which may be due to a small sample size. Another important issue is the content of cardiac consultations. Consultations are frequently limited to a statement of the increased surgical risk and make general recommendations concerning fluid balance, maintaining hemoglobin levels and continuing beta-blocker medication.

Stitgen et al. showed that 85% of patients were correctly screened according to the ACC/AHA guideline [6]. In our previous retrospective study on this matter (*n* = 388), we demonstrated 72% correctly screened patients [7] vs 87% in this study. The number of cardiac consultations has dropped to 23% in this study vs. 38% in our previous study. In addition, we found a marked decline in patients who were overscreened, 13% vs. 27% in our previous study. The most profound findings were in the intermediate risk group, where 24% of patients were not screened in accordance with the guidelines vs 54% in our previous study. The reason for incorrect preoperative cardiac screening in both our studies remained in > 90% of cases due to overscreening. We consider increased awareness of surgeons as well as anesthesiologists of the ACC/AHA guidelines and a reduced incidence of instant cardiac consultations requested by the physician at the emergency department for cardiac clearance are contributing to these findings. Sometimes the goal of preoperative screening seems to be to strive for ‘cardiac clearance’, some sort of cardiac approval that must be obtained before operation.

According to ACC/AHA guideline, patients who require non-invasive cardiac testing are those with active high risk cardiac conditions and those with intermediate risk clinical predictors combined with poor functional capacity. Whether a consultation is justified for patients in the intermediate risk group is decided by their functional capacity, measured by metabolic equivalent of task (METs), which is rather arbitrary. Currently, there exist no other clinical screening tools to identify those patients who need a cardiac consultation prior to hip fracture surgery.

It has been suggested to routinely perform an echocardiography in elderly patients with hip fractures [21]. Some authors have reported a benefit of routine echocardiography on mortality after hip fracture surgery [22], where others have not [23, 24]. Screening all hip fracture patients with transthoracic echocardiography (TTE) identified in 8% of patients significant aortic stenosis. Conversely severe aortic stenosis is no contraindication for surgery and does not influence early mortality [25, 26]. Another study found that preoperative TTE leads to an increased delay to surgery [9]. Delay to surgery is associated with worse outcome after hip fracture surgery [5, 23, 27–30]. Furthermore, TTE screening has cost implications, as this needs to be a continuously available service. There are no recommendations available from randomized controlled trials on the use of TTE screening in a hip fracture population. One retrospective matched-control study showed lower postoperative and 1-year mortality rates after focused TTE screening in a hip fracture population [22]. Unclear is whether these patients were only intermediate risk patients or also high risk patients and what their functional capacity was. Screening patients with a high chance of pathology is more logical than screening all hip fracture patients, therefore in the low risk group is TTE screening probably not useful and second not indicated by the ACC/AHA guideline.

A preoperative cardiac consultation rarely affects surgical management, but may influence anesthesiologic management, especially cardiovascular and fluid management after non-invasive testing with TTE in high risk or intermediate risk patients with low METs. The ACC/AHA guideline recommendation is level B evidence and is merely based on dobutamine stress echocardiography which is rarely used in the hip fracture population. Instead, bedside echocardiography without stress testing is performed to report on global cardiac and valvular function and volume status. The guideline states that non-invasive cardiac testing is reasonable for intermediate risk patients with < 4 METs if it will change patient management. As a result of this, TTE is probably most frequently overused in the intermediate risk group. Other indications for preoperative TTE are new developed dyspnoea without known aetiology or decompensated heart failure [31]. A study showed that TTE in accordance with the ACC/AHA guideline in only 14% of patients revealed disease with the potential to modify anesthesia or medical management [32]. If non-invasive cardiac testing like TTE prior to surgery is indicated, this should be performed without delay to surgery.

Not every form of anesthesia is equally suitable for every geriatric patient [33]. To improve outcome of high risk patients, some intraoperative strategies were studied in the literature. Continuous spinal anesthesia (CSA), with (minimally invasive) hemodynamic monitoring is suggested as alternative anesthesia in the case of severe aortic stenosis with minimal hemodynamic changes intraoperatively in hip fracture patients [34, 35]. CSA compared with combined spinal epidural anesthesia (CSE) showed better sensory blockade level and lower hemodynamic changes in 240 patients following major orthopaedic surgery [36]. According to a Cochrane systematic review, other advanced hemodynamic monitoring strategies, such as esophageal Doppler monitoring, goal-directed hemodynamic treatment (with LiDCO monitor) or Swan Ganz pulmonary-artery catheter in comparison with standard care and conventional fluid management did not show improvement on postoperative outcome after hip fracture surgery [37].

Respiratory and cardiovascular complications occurred significantly more often in the high risk group in comparison with the intermediate and low risk group and increased the risk for in-hospital death fivefold to a 30-day mortality rate of 31%. Another study found that pneumonia and heart failure after hip fracture surgery lead to a mortality rate of 43% and 65%, respectively [38]. Three or more co-morbidities, respiratory disease and malignancy were preoperative variables that were significantly related to increased 30-day mortality. In a multivariate regression analysis, we showed that a previous cardiac history was a predictor for early and late mortality. The increased early mortality in the cardiac history group could be explained by postoperative respiratory and cardiovascular complications and the increased late mortality could be explained by the effect of comorbidity on the long-term. We did not examine whether patients with a cardiac history had equal recovery chances compared to patients without a cardiac history.

Mortality after hip fracture remains high and extremely high for high risk patients. Hip fractures are associated with an in-hospital mortality rate of 7–14%, reaching up to 36% within 1 year of surgery [7, 39–43]. Over the past 3 decades, mortality rates after hip fracture surgery hardly changed. One year mortality for intertrochanteric fractures remained about 23% after 1999 [44]. Although a steady decrease was found in the UK, 11% 30-day mortality in 2003 decreased to 8% in 2013 [45]. A combined number of improvements of care, including the implementation of fast-track care pathways, input from orthogeriatricians, quick patient medical optimization, early surgery and advanced rehabilitation protocols have contributed to this [33, 45].

In conclusion, preoperative cardiac screening in adherence to the ACC/AHA guideline is associated with a diminished use of preoperative resources. Overscreening leads to greater delay to surgery, which poses a risk for perioperative complications and early mortality.
